# Sports participation and health-related quality of life: a longitudinal observational study in children

**DOI:** 10.1007/s11136-019-02219-4

**Published:** 2019-06-03

**Authors:** Janet Moeijes, Jooske T. van Busschbach, Ruud J. Bosscher, Jos W. R. Twisk

**Affiliations:** 1 Department of Human Movement and Education, Windesheim University of Applied Sciences, Campus 2-6, 8017 CA Zwolle, The Netherlands; 2 Department of Epidemiology and Biostatistics, Amsterdam Public Health Research Institute, Amsterdam UMC, Vrije Universiteit Amsterdam, Van der Boechorststraat 7, 1081 BT Amsterdam, The Netherlands; 3 University Center for Psychiatry, University Medical Center Groningen, University of Groningen, P.O. Box 30001, 9700 RB Groningen, The Netherlands

**Keywords:** Children, Frequency of sports participation, Outdoor sports, Team sports, Health-related quality of life, Longitudinal associations

## Abstract

**Purpose:**

In this study, longitudinal associations between sports participation and health-related quality of life (HRQoL) were explored. Sports participation was operationalized as membership of a sports club, frequency of sports participation, performing individual versus team sports and performing indoor versus outdoor sports. The concept of HRQoL referred to the self-perceived enjoyment and satisfaction with one’s personal health situation.

**Methods:**

Data from 618 fourth-grade primary school children were included at baseline; 10–13 months later, 417 children (response rate 67.5%) were retained. At both time points, children reported on sports participation (Move and Sports Monitor Questionnaire—youth aged 8–12 years) and health-related quality of life (KIDSCREEN-52). Because of the clustering of children in schools, data were analysed using linear mixed models. Analyses were adjusted for sex, age, BMI, household composition, SES and frequency of sports participation.

**Results:**

The questionnaires were fully completed by 417 children. High sports-active children showed better scores on almost all dimensions of HRQoL than moderate [difference (*B*) = − 1.82 (*p* = 0.05) to − 1.51 (*p* = 0.05)] or low ports-active children [difference (*B*) = − 3.67 (*p* < 0.001) to − 1.95 (*p* = 0.03)] and non-sports club members [difference (*B*) = − 5.58 (*p* < 0.001) to – 2.65 (*p* = 0.02)]. Unlike frequency, the other examined characteristics of sports participation were only to a limited extent longitudinal associated with HRQoL.

**Conclusion:**

As frequency is more relevant than the form of sports participation, children should be encouraged to perform any kind of sports activity on a very regular base.

**Electronic supplementary material:**

The online version of this article (10.1007/s11136-019-02219-4) contains supplementary material, which is available to authorized users.

## Introduction

Health-related quality of life (HRQoL) is an essential outcome measure for the evaluation of health care programs and services [1, 2]. This multidimensional construct covers “physical, emotional, mental, social, and behavioural components of well-being and functioning as perceived by patients and/or other individuals” [3]. Whereas psychosocial health relates to a person’s actual health situation, the concept of HRQoL pertains to his or her self-perceived well-being and functioning [4].

Recent systematic reviews suggest that HRQoL is positively associated with sports participation. With one review focussing on adults [5], three other recent reviews present an overview of research on children (aged twelve and younger, in primary education) and adolescents (thirteen and older, in high school). Eime et al. [6] indicated that sports participation, especially performing club-based sports, may lead to better HRQoL in the broad age group of children and adolescents. They found a stronger association for participation in team sports compared to participation in individual sports. Wu et al. [7] concluded from their systematic review that children and adolescents with a higher frequency of physical activity show better HRQoL. The systematic review and meta-analysis by Marker et al. [8] identified the same but relatively weak relationship between physical activity and better HRQoL in children and adolescents.

In a majority of studies included in recent reviews on children and adolescents, physical activity as such is emphasized, ignoring whether or not children and adolescents were active members of a sports club or had a more active life-style in general. Contrary to the studies reviewed by Eime et al. [6], all studies reviewed by Marker et al. [8] and the majority of studies reviewed by Wu et al. [7] did not reflect on sports participation as a separate variable in their analyses.

Of those studies aimed at sports participation, most focused on samples of adolescents, for instance high school populations [9–16] and only few studied sports participation in children in the age of twelve and younger [17–21]. Moreover, although some of the reviewed studies on children use the concept of emotional or social well-being [17, 18], the health aspects investigated in these studies relate more to a child’s actual health situation than to his or her evaluation of this state. So, these studies paid more attention to the psychosocial health of children than to their HRQoL.

All in all, there are three studies that aimed to study the relationship between sports participation and HRQoL in children of twelve and younger [19–21]. Two of these three studies, Sánchez-López et al. [19] and Tsiros et al. [20] limit their scope to membership of a sports club and/or frequency of sports participation. In their conclusion, Tsiros et al. [20] did recommend to pay attention to a potential relationship between performing indoor versus outdoor sports and HRQoL. Only Vella et al. [21] took the distinction between team and individual sports into account among various other characteristics of sports participation. Their study confirmed that not only membership of a sports club but also more specifically performing team sports instead of individual sports were associated with better HRQoL.

In line with Coalter [22] and Marker et al. [8] who stated that progress in this field can be made by focussing future research on specific characteristics of sports participation, the present study explored longitudinal associations between various characteristics of sports participation and different dimensions of HRQoL. It concerned associations between membership of a sports club, frequency of sports participation, performing individual versus team sports and performing indoor versus outdoor sports on the one hand and ten dimensions of HRQoL on the other.

As noted by Marker et al. [8], most descriptive studies on the relationship between sports participation and HRQoL have a cross-sectional design. The present study is, however, aimed at exploring longitudinal associations.

## Methods

### Study design and participants

This national-representative study examined sports participation and HRQoL in fourth- and fifth-grade primary school children in the Netherlands. The study comprised a cross-sectional component to be published separately, and a longitudinal component with data gathering repeated after a period of 10–13 months reported here. Data were collected from November 2011 to June 2014, mostly in autumn and spring.

The primary schools used in the present study were spread fairly well in terms of geographical location (eight of the 12 provinces) and urbanization rate (14 schools in rural areas and 15 schools in urban areas). As a result, the group of children included in the longitudinal part of the study was comparable with the general Dutch population of primary school children for neighbourhood socioeconomic status (SES), i.e. 6.4% of the children in the sample had a low SES background, while 5.5% of all Dutch children lived in low SES families [23]. Body mass index (BMI), i.e. BMI by underweight and BMI by overweight were respectively 6.4 and 13.1% in the sample versus 6.6 and 11.6% in the population [24] and for sports participation (in terms of membership in a sports club) with 87% in the sample versus 83% in the general population [25].

The choice to test children in the fourth and then a year later in the fifth grade was based on the fact that children of 10–12 are old enough to independently complete questionnaires. This is a requisite for adequate self-report on HRQoL that significantly differs from reporting by parents or guardians [26]. Furthermore, these children were young enough not to have entered puberty in which physical and psychological changes [27, 28] may overshadow possible associations between sports participation and HRQoL.

Primary schools were first addressed by phone and email. If a school director replied to be interested in the research, an email with detailed information was sent, and he or she was again contacted by phone 1 week later. After the school director had given permission by phone for research activities at this school, he or she supplied written information about the aim, nature and practice of the research to the parents and eventual guardians of children. Written informed consent from parent or guardian was a prerequisite to participate in this research.

Of the 73 schools involved in the cross-sectional part the study, 29 schools also participated in the longitudinal part. Most of these schools (21) were involved twice in the longitudinal part of the study. Some schools (eight) participated three times (for more information see Supplementary material—Additional file 1: Flowchart gathering schools). Headmasters gave permission for each occasion.

At the initial meeting, children were given a booklet containing questionnaires which were piloted in a small test sample of 43 children in three primary schools. The questionnaires had to be completed individually in the classroom, whereby children could consult their classroom teacher if assistance was needed. The questionnaires were collected 1 week later. Subsequently, anthropometric data were obtained during school time.

**Fig. 1:**
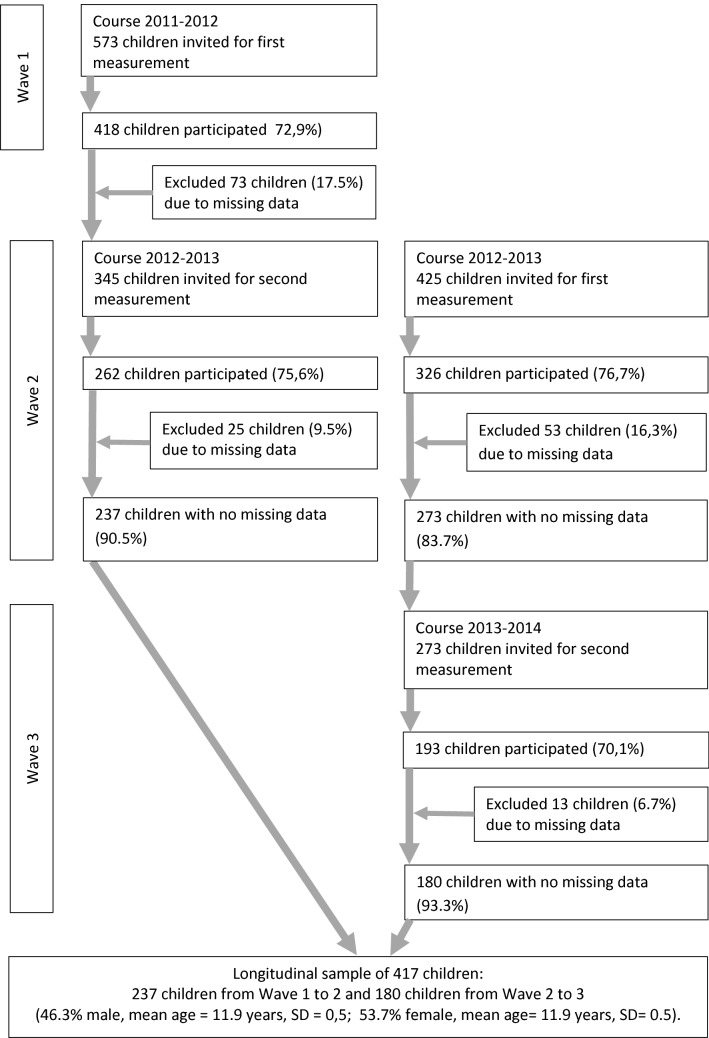
Flowchart regarding the gathering of children

Figure 1 presents information about the sample recruitment and selection for this longitudinal study.

The study was conducted in accordance with the guidelines proposed by the World Medical Association Declaration of Helsinki and was approved by the Medical Ethical Committee at VU University Medical Center Amsterdam (12/151). All parents and guardians of children in the sample provided a written informed consent.

## Measures

### HRQoL

HRQoL was measured using the KIDSCREEN-52, a self-reported generic measure of HRQoL in children and adolescents aged between 8 and 18 years [3, 29]. We used a Dutch version of the original English version of the questionnaire. The Dutch version was obtained by translating the original version according to a standardized methodology based on international cross-cultural translation guidelines [30, 31].

The KIDSCREEN-52 includes 52 items covering ten dimensions which are classified into three domains. The ‘physical well-being’ dimension relates to the physical domain and includes five items, e.g. the item ‘In general, how would you say your health is?’. The psychological domain comprises the dimensions ‘psychological well-being’ (six items, e.g. ‘Thinking about the last week…Has your life been enjoyable?), ‘moods and emotions’ (seven items, e.g. ‘Thinking about the last week…Have you felt sad?’), and ‘self-perception’ dimension (five items, e.g. ‘Thinking about the last week …Have you been happy with the way you are?’). The social domain relates to the dimensions ‘autonomy’ (5 items, e.g. ‘Thinking about the last week…Have you had enough time for yourself?’), ‘parent relations and home life’ (six items, e.g. ‘Thinking about the last week…Have your felt loved by your parent(s)?’), ‘social support and peers’ (six items, e.g. ‘Thinking about the last week…Have you had fun with your friends?’), ‘school environment’ (six items, e.g. ‘Thinking about the last week … Have you been happy at school?’), ‘social acceptance (bullying)’ (three items’, e.g. Thinking about the last week Have other girls and boys bullied you?’), and ‘financial resources’ (three items, e.g. ‘Thinking about the last week…Have you had enough money for your expenses?’).

All items are rated on a five-point Likert scale (ranging from ‘‘never’’ to ‘‘always’’ or from ‘‘not at all’’ to ‘‘extremely’’). After recoding some of the items, a higher score indicates better HRQoL.

The KIDSCREEN-52 has a satisfactory internal consistency with Cronbach’s alphas for the ten dimensions ranging from 0.77 to 0.89 [3, 32]. The test–retest reliability is also satisfying with intraclass correlation coefficients (ICCs) ranging from 0.56 to 0.77 [3, 32]. The questionnaire shows good results in terms of convergent, known groups’ and criterion validity [32].

### Sports participation

The study focused on sports activities in sports clubs, the primary environment in which Dutch children participate in sports [33]. A child’s participation in sports club activities was measured using two items from the self-report Move and Sports Monitor Questionnaire (MSMQ)—youth aged 8–12 years [34]: membership in a sports club and frequency of training and matches per week. Frequency of sports participation was classified into tertiles. We added a third question about the sport(s) in which a child participated, which made it possible to determine whether the child practised individual and/or team sports and whether he or she was involved in indoor and/or outdoor sports. As described in Moeijes et al. [35], the validation of the questions about sports participation took place in a pilot study among primary school children, an expert meeting, individual expert consultations and a comparison of some research findings about children’s sports participation with national data. See for more information Supplementary material—Additional file 2: Validation process sports participation questions.

### Potential confounders

The covariates of age (continuous), sex (dichotomous), BMI (continuous), household composition (dichotomous) and SES (continuous) were used in all analyses. According to literature, these factors are associated with HRQoL [1, 36–38]. Frequency of sports participation (continuous) was used as a covariate in the analyses of the associations between performing individual versus team sports and indoor versus outdoor sports on the one hand and HRQoL on the other. These associations were analysed given a certain frequency of sports participation to avoid the risk of finding spurious associations or overlooking important associations.

Parents or guardians reported the child’s date of birth and sex. Researchers who visited the schools measured height and weight. Height was measured by the Seca 201 or 203 system (Basel, Switzerland) and weight by the Seca Sensa 804 (Basel, Switzerland) or the Tanita BC 601 digital scale (Tokyo, Japan). BMI was calculated as weight divided by height squared (kg/m^2^) [39]. Household composition was measured as living in a two-parent family or another type of household, for example a one-parent family [40]. The SES of the child’s parents or guardians was based on postcodes and the status score per postcode derived from the Dutch Social and Cultural Planning Office in 2010 [41].

### Statistical analyses

As prescribed in the KIDSCREEN manual, Rasch scores were calculated for each dimension of HRQoL. These scores were transformed into *T*-values with a mean of 50 and a standard deviation (SD) of 10 [3].

Children who had missing KIDSCREEN-52 data were excluded to conform to the procedure outlined in the manual. The scores on the 5–7-items dimensions were only calculated if not more than one item of the dimension was left unanswered. The scores on the three-items dimension were only calculated if all questions were answered. A greater number of missing data resulted in the exclusion of the child from the analyses [3]. Children who failed to answer one or more questions concerning the sports participation questionnaire MSMQ, BMI, SES or household composition, were excluded from the analyses.

Data for the children who did not participate in the second measurement were compared with the data for the children who participated in both measurements using independent *t* tests (for continuous variables) or *χ*2 tests (for dichotomous and categorical variables).

The longitudinal associations between each of the characteristics of sports participation and each of the ten outcome variables separately were analysed using linear mixed models, taking into account the three-level structure of the data: repeated measures (level 1) were clustered within children (level 2) and children were clustered within the schools (level 3). For the outcome variables (i.e. the ten dimensions of the KIDSCREEN-52), multilevel models included random intercepts for the children and the schools. Before analyses, the normality of the distribution of the outcome variables was determined by visual examination of histograms.

In addition to crude analyses, analyses were performed adjusting for sex, age, BMI, household composition, SES and frequency of sports participation. Frequency of sports participation as a potential confounder co-varied moderately with performing individual versus team sports (*ρ* = 0.59, *p* < 0.001) and performing indoor versus outdoor sports (*ρ* = 0.63, *p* < 0.001). The other potential confounders co-varied with characteristics of sports participation only slightly. Thus, there is no multicollinearity among the covariates and the independent variables.

All data were analysed using IBM SPSS (version 24, IBM, New York, USA). For all analyses, a two-tailed significance level of *p* < 0.05 was considered statistically significant.

## Results

A number of 618 fourth-grade primary school children filled in the questionnaires in the first measurement. The majority of these children (417, response rate 67.5%) fully completed the questionnaires in the second measurement and were included in the study. The 201 excluded fifth-graders (32.5%) did not participate in the second measurement because they were no longer interested (163 children), or were excluded due to missing data (38 children of whom 1, 3 and 34 had missing data on sports participation characteristics, BMI and HRQoL, respectively).

The group of children who only supplied information for the first measurement differed significantly from the group of children who were measured twice. Those who dropped out of the study were more active (frequency of sports participation: 2.73 vs. 2.45; *p* = 0.05) and had lower scores on physical well-being (54.71 vs. 56.72 points, *p* = 0.01) than those who remained in the study. No other significant differences were found. Table 1 shows descriptive information of the sample under study.

**Table 1:** Demographic characteristics of the sample at baseline (*N* = 618) and follow-up (*N* = 417)

	Fourth grade				Fifth grade			
	*N*	%	Mean	SD	*N*	%	Mean	SD
Sex								
Boys	288	46.6			193	46.3		
Girls	330	53.4			224	53.7		
Age			11.0	0.5			11.9	0.5
BMI			17.7	2.4			18.2	2.3
SES			0.25	0.92			0.29	0.89
Household composition								
Two parent family	503	81.4			328	78.7		
Others	115	18.6			89	21.3		
Membership sports club								
No	76	12.3			56	13.4		
Yes	542	87.7			361	86.6		
Frequency of sports participation			2.48	1.65			2.56	1.56
Non-members	76	12.3			56	13.4		
Low sports-actives^a^	210	34.0			117	28.1		
Moderate sports-actives^b^	171	27.7			145	34.8		
High sports-actives^c^	161	26.1			99	23.7		
Individual versus team sports								
Non-members	76	12.3			56	13.4		
Individual sports	143	23.1			93	22.3		
Team sports	297	48.1			210	50.4		
Individual as well as team sports	102	16.5			58	13.9		
Indoor versus outdoor sports								
Non-members	76	12.3			56	13.4		
Indoor sports	186	30.1			114	27.3		
Outdoor sports	277	44.8			208	49.9		
Indoor as well as outdoor sports	79	12.8			39	9.4		
Kidscreen-52								
Physical domain Physical well-being			56.07	9.37			56.83	9.93
Psychological domain Psychological well-being			54.84	8.65			56.48	9.23^d^
Moods and emotions			51.50	10.16			54.41	10.71^d^
Self-perception			55.23	9.28			56.18	9.92
Social domain autonomy			54.48	8.62			56.28	9.10^d^
Parent relation and home life			56.08	8.41			57.14	8.77^d^
Social support and peers			53.53	9.16			56.12	9.42^d^
School environment			56.74	8.94			57.74	8.95
Social acceptance (bullying)			49.09	10.43			50.43	9.92^d^
Financial resources			53.33	9.27			55.57	8.52^d^

^a^Low sports-actives: 1–2.2 times a week

^b^Moderate sports-actives: 2.25–3 times a week

^c^High sports-actives: 3.02–9 times a week

^d^Scores 1st and 2nd measurement significantly differ

### Membership sports club

According to the analyses adjusted for gender, age, BMI, SES and household composition, membership of a sports club was significantly longitudinally associated with better HRQoL in the physical domain (dimension ‘physical well-being’, *B* = 3.02, *p* = 0.002), to a lesser extent in the social domain (dimension ‘social support and peers’, *B* = 2.10, *p* = 0.03; ‘financial resources’, *B* = 2.15, *p* = 0.02), and not at all in the psychological domain (Table 2).

**Table 2:** Associations between membership of a sports club and HRQOL dimensions

	Crude analyses			Adjusted analyses^a^		
	*B* ^b^	*p*	CI	*B* ^b^	*p*	CI
Physical domain						
Physical well-being						
Non-member	Reference group					
Member	3.72	**< 0.001**	1.77–5.67	3.20	**0.002**	1.25–5.14
Psychological domain						
Psychological well-being						
Non-member	Reference group					
Member	0.58	0.53	− 1.23 to 2.40	0.47	0.62	− 1.36 to 2.29
Moods and emotions						
Non-member	Reference group					
Member	− 0.30	0.78	− 2.43 to 1.82	− 0.36	0.74	− 2.48 to 1.77
Self-perception						
Non-member	Reference group					
Member	0.47	0.64	− 1.49 to 2.43	− 0.02	0.98	− 1.96 to 1.91
Social domain						
Autonomy						
Non-member	Reference group					
Member	0.48	0.61	− 1.35 to 2.30	0.37	0.69	− 1.45 to 2.20
Parents and home life						
Non-member	Reference group					
Member	1.27	0.15	− 0.47 to 3.02	1.16	0.19	− 0.59 to 2.90
Social support and peers						
Non-member	Reference group					
Member	2.18	**0.03**	0.24–4.12	2.10	**0.03**	0.15 to 3.01
Social acceptance (bullying)						
Non-member	Reference group					
Member	1.27	0.24	− 0.83 to 3.38	1.13	0.30	− 0.99 to 3.26
School environment						
Non-member	Reference group					
Member	− 0.14	0.88	− 1.94 to 1.66	− 0.04	0.97	− 1.86 to 1.78
Financial resources						
Non-member	Reference group					
Member	2.29	**0.01**	0.47–4.11	2.15	**0.02**	0.32–3.97

Significant p’s are in bold

^a^Adjusted for gender, age, BMI, SES, and household composition

^b^Unstandardized regression coefficient

### Frequency of sports participation

The analyses adjusted for the five before-mentioned covariates show that high and moderate sports-active children significantly had better HRQoL than low sports-active children and non-sports club members in the physical domain (dimension ‘physical well-being’, *B* = − 3.67, *p* < 0,001 and *B* = − 1.94, *p* = 0.02, respectively, for low sports-actives; *B* = − 5.58, *p* < 0.001 and *B* = − 3.81, *p* < 0.001, respectively, for non-sports club members).

For the psychological domain, high and moderate sports-active children showed better HRQoL than low sports-active children for the dimension ‘psychological well-being’ (*B* = − 3.53, *p* < 0.001 and *B* = − 2.07, *p* = 0.01, respectively). Furthermore, high sports-active children showed better HRQoL than non-sports club members for the dimension ‘psychological well-being’ (*B* = − 2.65, *p* = 0.02) and showed better HRQoL than moderate and low sports-actives for the dimension ‘moods and emotions’ (*B* = − 1.82, *p* = 0.05 and *B* = − 2.36, *p* = 0.02, respectively). For the dimension ‘self-perception’, high sports-active children showed better HRQoL than low sports-active children (*B* = − 1.95, *p* = 0.03).

In the social domain, high sports-active children showed better HRQoL than moderate and low sports-active children and non-sports club members for the dimension ‘parents and homelife’ (*B* = − 1.51, *p* = 0.05, *B* = − 2.54, *p* = 0.002 and *B* = − 2.90, *p* = 0.01, respectively). Finally, for the dimension ‘social support and peers’, high sports-active children showed better HRQoL than low sports-active children and non-sports club members (*B* = − 2.97, *p* = 0.001 and *B* = − 4.00, *p* < 0.001, respectively) (Table 3).

**Table 3:** Associations between frequency of sports participation and HRQOL dimensions

	Crude analyses			Adjusted analyses^a^		
	B^b^	p	CI	B^b^	p	CI
Physical domain						
Physical well-being						
High sports-active	Reference group					
Moderate sports-active	− 1.55	0.07	− 3.23 to 0.13	− 1.74	**0.04**	− 3.41 to − 0.06
Low sports-active	− 3.98	**< 0.001**	− 5.75 to − 2.20	− 3.67	**< 0.001**	− 5.46 to − 1.89
Non-members	− 6.16	**< 0.001**	− 8.44 to − 3.89	− 5.58	**< 0.001**	− 7.86 to − 3.30
Psychological domain						
Psychological well-being						
High sports-active	Reference group					
Moderate sports-active	− 1.25	0.12	− 2.82 to 0.31	− 1.44	0.07	− 3.00 to 0.13
Low sports-active	− 3.61	**< 0.001**	− 5.25 to − 1.97	− 3.53	**< 0.001**	− 5.18 to − 1.87
Non-members	− 2.66	**0.01**	− 4.78 to − 0.53	− 2.65	**0.02**	− 4.79 to − 0.51
Moods and emotions						
High sports-active	Reference group					
Moderate sports-active	− 1.51	0.11	− 3.34 to 0.33	− 1.82	**0.05**	− 3.64 to 0.00
Low sports-active	− 2.72	**0.01**	− 4.66 to − 0.78	− 2.36	**0.02**	− 4.31 to − 0.41
Non-members	− 1.51	0.24	− 4.03 to 1.01	− 1.42	0.27	− 3.95 to 1.10
Self-perception						
High sports-active	Reference group					
Moderate sports-active	− 0.64	0.46	− 2.34 to 1.06	− 1.03	0.23	− 2.71 to 0.64
Low sports-active	− 2.42	**0.01**	− 4.21 to − 0.63	− 1.95	**0.03**	− 3.73 to − 0.17
Non-members	− 1.85	0.12	− 4.16 to 0.47	− 1.25	0.28	− 3.54 to 1.03
Social domain						
Autonomy						
High sports-active	Reference group					
Moderate sports-active	− 0.50	0.55	− 2.09 to 1.11	− 0.74	0.36	− 2.34 to 0.85
Low sports-active	− 1.52	0.8	− 3.20 to 0.16	− 1.26	0.15	− 2.95 to 0.43
Non-members	− 1.33	0.23	− 3.48 to 0.82	− 1.21	0.27	− 3.37 to 0.95
Parents and home life						
High sports-active	Reference group					
Moderate sports-active	− 1.30	0.09	− 2.81 to 0.20	− 1.51	**0.05**	− 3.01 to − 0.01
Low sports-active	− 2.64	**0.001**	− 4.23 to − 1.06	− 2.54	**0.002**	− 4.13 to − 0.94
Non-members	− 2.95	**0.01**	− 5.01 to − 0.89	− 2.90	**0.01**	− 4.97 to − 084
Social support and peers						
High sports-active	Reference group					
Moderate sports-active	− 1.36	0.12	− 3.05 to 0.34	− 1.53	0.08	− 3.22 to 0.15
Low sports-active	− 2.91	**0.001**	− 4.69 to − 1.14	− 2.97	**0.001**	− 4.75 to − 1.18
Non-members	− 3.91	**0.001**	− 6.19 to − 1.63	− 4.00	**0.001**	− 6.29 to − 1.71
Social acceptance (bullying)						
High sports-active	Reference group					
Moderate sports-active	− 0.05	0.96	− 1.88 to 1.79	− 0.16	0.86	− 2.00 to 1.67
Low sports-active	− 1.95	**0.05**	− 3.88 to − 0.03	− 1.85	0.06	− 3.80 to 0.10
Non-members	− 2.17	0.09	− 4.66 to 0.31	− 2.08	0.11	− 4.60 to 0.44
School environment						
High sports-active	Reference group					
Moderate sports-active	− 0.25	0.76	− 1.82 to 1.33	− 0.17	0.83	− 1.75 to 1.41
Low sports-active	− 1.23	0.15	− 2.89 to 0.43	− 1.49	0.08	− 3.17 to 0.20
Non-members	− 0.50	0.65	− 2.63 to 1.64	− 0.72	0.51	− 2.87 to 1.44
Financial resources						
High sports-active	Reference group					
Moderate sports-active	− 1.12	0.17	− 2.70 to 0.48	− 1.29	0.11	− 2.87 to 0.29
Low sports-active	− 1.48	0.08	− 3.15 to 0.18	− 1.28	0.14	− 2.96 to 0.41
Non-members	− 3.36	**0.002**	− 5.51 to 1.21	− 3.19	**0.004**	− 5.36 to − 0.29

Significant p’s are in bold

^a^Adjusted for gender, age, BMI, SES, and household composition

^b^Unstandardized regression coefficient

### Performing individual versus team sports

According to the analyses adjusted for the five abovementioned covariates and frequency of sports participations, children participating in team sports showed significantly worse HRQoL on the dimension ‘moods and emotions’ compared to children participating in individual sports (*B* = − 2.22, *p* = 0.03). No other significant associations were observed (Table 4).

**Table 4:** Associations between performing individual versus team sports and HRQOL dimensions

	Crude analyses			Adjusted^a^			Adjusted^b^		
	B^c^	p	CI	B^c^	p	CI	B^c^	p	CI
Physical domain									
Physical well-being									
Individual sports	Reference group								
Team sports	0.48	0.59	− 1.32 to 2.28	0.09	0.92	− 1.71 to 1.90	− 0.44	0.63	− 2.23 to 1.35
Individual as well as team sports	1.09	0.34	− 1.16 to 3.34	1.29	0.26	− 0.94 to 3.53	− 0.53	0.66	− 2.88 to 1.83
Non-members	− 3.31	**0.004**	− 5.58 to − 1.04	− 2.96	**0.01**	− 5.22 to − 0.70	− 0.15	0.91	− 2.73 to 2.43
Psychological domain									
Psychological well-being									
Individual sports	Reference group								
Team sports	0.54	0.53	− 1.14 to 2.22	0.57	0.52	− 1.14 to 2.27	0.10	0.91	− 1.60 to 1.80
Individual as well as team sports	0.90	0.40	− 1.18 to 2.98	1.29	0.22	− 0.79 to 3.37	− 0.30	0.79	− 2.51 to 1.92
Non-members	− 0.16	0.88	− 2.28 to 1.96	0.03	0.98	− 2.10 to 2.15	2.36	0.06	− 0.07 to 4.78
Moods and emotions									
Individual sports	Reference group								
Team sports	− 0.67	0.09	− 3.70 to 0.27	− 1.94	0.06	− 3.95 to 0.08	− 2.22	**0.03**	− 4.24 to − 0.20
Individual as well as team sports	− 0.42	0.74	− 2.87 to 2.03	− 0.06	0.96	− 2.50 to 2.38	− 1.15	0.39	− 3.76 to 1.45
Non-members	− 1.72	0.60	− 3.15 to 1.81	− 0.65	0.61	− 3.11 to 1.82	0.99	0.49	− 1.83 to 3.81
Self-perception									
Individual sports	Reference group								
Team sports	− 1.01	0.28	− 2.82 to 0.82	− 1.60	0.08	− 3.39 to 0.19	− 1.77	0.05	− 3.56 to 0.03
Individual as well as team sports	− 0.01	1.00	− 2.26 to 2.25	0.31	0.78	− 1.89 to 2.53	− 0.30	0.80	− 2.66 to 2.05
Non-members	− 1.01	0.39	− 3.30 to 1.28	− 0.77	0.50	− 3.01 to 1.48	0.57	0.66	− 2.01 to 3.15
Social domain									
Autonomy									
Individual sports	Reference group								
Team sports	− 0.62	0.46	− 2.28 to 1.04	− 0.77	0.37	− 2.45 to 0.91	0.92	0.29	− 2.61- 0.77
Individual as well as team sports	− 0.63	0.55	− 2.73 to 1.46	− 0.21	0.84	− 2.30 to 1.88	− 0.72	0.53	− 2.95 to 1.52
Non-members	− 0.91	0.40	− 3.04 to 1.22	− 0.82	0.45	− 2.94 to 1.31	− 0.02	0.99	− 2.48 to 2.44
Parents and home life									
Individual sports	Reference group								
Team sports	− 0.93	0.26	− 2.53 to 0.71	− 0.97	0.25	− 2.61 to 0.67	− 1.33	0.11	− 2.97 to 0.30
Individual as well as team sports	− 0.34	0.74	− 2.34 to 1.67	0.04	0.97	− 1.96 to 2.04	− 1.26	0.25	− 3.40 to 0.87
Non-members	− 1.82	0.08	− 3.79 to 0.28	− 1.66	0.11	− 3.68 to 0.37	0.28	0.81	− 2.04 to 2.60
Social support and peers									
Individual sports	Reference group								
Team sports	− 0.91	0.31	− 2.67 to 0.85	− 0.73	0.42	− 2.53 to 1.06	− 1.09	0.23	− 2.89 to 0.71
Individual as well as team sports	0.17	0.88	− 2.06 to 2.39	0.56	0.62	− 1.67 to 2.78	− 0.71	0.56	− 3.08 to 1.67
Non-members	− 2.64	**0.02**	− 4.90 to − 0.37	− 2.39	**0.04**	− 4.66 to − 0.13	− 0.48	0.72	− 3.09 to 2.12
Social acceptance (bullying)									
Individual sports	Reference group								
Team sports	− 0.41	0.70	− 2.36 to 1.53	− 0.46	0.65	− 2.44 to 1.53	− 0.67	0.51	− 2.57 to 1.36
Individual as well as team sports	0.90	0.47	− 1.53 to 3.32	1.08	0.38	− 1.35 to 3.52	0.29	0.83	− 1.93 to 3.20
Non-members	− 1.35	0.28	− 3.81 to 1.10	− 1.20	0.34	− 3.68 to 1.27	− 0.03	0.98	− 3.07 to 2.52
School environment									
Individual sports	Reference group								
Team sports	− 1.77	**0.04**	− 3.42 to − 0.12	− 1.44	0.09	− 3.12 to 0.24	− 1.57	0.07	− 3.27 to 0.11
Individual as well as team sports	0.00	1.00	− 2.07 to 2.07	0.22	0.84	− 1.86 to 2.30	− 0.31	0.78	− 2.55 to 1.91
Non-members	− 0.81	0.45	− 2.91 to 1.28	− 0.68	0.53	− 2.79 to 1.43	0.15	0.90	− 2.29 to 2.59
Financial resources									
Individual sports	Reference group								
Team sports	− 0.97	0.25	2.64 to 0.70	− 0.94	0.28	− 2.64 to 0.76	− 1.16	0.18	− 2.87 to 0.55
Individual as well as team sports	− 0.53	0.62	2.62 to 1.56	− 0.23	0.83	− 2.33 to 1.86	− 1.05	0.36	− 3.29 to 1.19
Non-members	− 2.89	**0.01**	− 5.02 to − 0.77	− 2.67	**0.01**	− 4.80 to − 0.54	− 1.43	0.25	− 3.88 to 1.02

Significant p’s are in bold

^a^Adjusted for gender, age, BMI, SES and household composition

^b^Adjusted for gender, age, BMI, SES, household composition and frequency

^c^Unstandardized regression coefficient

### Performing indoor versus outdoor sports

The analyses adjusted for frequency of sports participation, and the other five covariates show that children performing outdoor sports had a significantly more favourable HRQoL in the psychological domain (dimension ‘self-perception’; *B* = 1.82, *p* = 0.05) and the social domain (dimension ‘financial resources’; *B* = 2.36, *p* = 0.01) compared to children performing indoor sports. Children performing indoor sports as well as outdoor sports showed also a better HRQoL in for the dimension ‘self-perception’ (*B* = 3.26, *p* = 0.01) than children performing indoor sports. There was no significant difference observed for the physical domain (Table 5).

**Table 5:** Associations between performing indoor versus outdoor sports and HRQOL dimensions

	Crude analyses			Adjusted analyses^a^			Adjusted analyses^b^		
	B^c^	p	CI	B^c^	p	CI	B^c^	p	CI
Physical domain									
Physical well-being									
Indoor sports	Reference group								
Outdoor sports	3.26	**< 0.001**	1.56 to 4.95	2.54	**0.01**	0.77 to 4.31	1.75	0.05	− 0.03 to 3.54
Indoor as well as outdoor sports	2.67	**0.03**	0.24 to 5.09	2.86	**0.02**	0.45 to 5.27	1.08	0.41	− 1.48 to 3.64
Non-members	− 1.83	0.10	− 4.01 to 0.34	− 1.69	0.13	− 3.86 to 0.48	0.65	0.60	− 1.82 to 3.13
Psychological domain									
Psychological well-being									
Indoor sports	Reference group								
Outdoor sports	2.04	**0.01**	0.45 to 3.62	1.80	**0.03**	0.13 to 3.47	1.01	0.24	− 0.69 to 2.71
Indoor as well as outdoor sports	0.95	0.41	− 1.33 to 3.23	1.17	0.32	− 1.11 to 3.44	− 0.53	0.67	− 2.95 to 1.89
Non-members	0.54	0.60	− 1.50 to 2.57	0.50	0.63	− 1.54 to 2.54	2.68	**0.02**	0.36 to 5.00
Moods and emotions									
Indoor sports	Reference group								
Outdoor sports	2.63	**0.01**	0.75 to 4.53	2.21	**0.03**	0.22–4.20	1.79	0.09	− 0.25 to 3.83
Indoor as well as outdoor sports	2.11	0.12	− 0.58 to 4.79	2.33	0.09	− 0.34 to 5.00	1.44	0.32	− 1.42 to 4.31
Non-members	1.83	0.13	− 0.55 to 4.22	1.63	0.18	− 0.75 to 4.01	2.76	**0.05**	0.04 to 5.47
Self-perception									
Indoor sports	Reference group								
Outdoor sports	3.13	**< 0.001**	1.42 to 4.84	1.97	**0.03**	0.22 to 3.73	1.82	**0.05**	0.01 to 3.62
Indoor as well as outdoor sports	3.37	**0.01**	0.92 to 5.82	3.80	**0.002**	1.40 to 6.19	3.26	**0.01**	0.88 to 6.03
Non-members	1.45	0.19	− 0.73 to 3.64	1.39	0.20	− 0.76 to 3.55	1.84	0.15	− 0.64 to 4.32
Social domain									
Autonomy									
Indoor sports	Reference group								
Outdoor sports	0.54	**0.03**	0.20 to 3.34	1.33	0.11	− 0.32 to 2.98	1.19	0.17	− 0.50 to 2.89
Indoor as well as outdoor sports	1.03	0.37	− 1.24 to 3.30	1.33	0.25	− 0.93 to 3.59	1.01	0.41	− 1.42 to 3.45
Non-members	1.77	0.60	− 1.50 to 2.59	0.41	0.69	− 1.63 to 2.46	0.83	0.49	− 1.53 to 3.20
Parents and home life									
Indoor sports	Reference group								
Outdoor sports	1.97	**0.01**	0.42 to 3.51	0.69	**0.05**	0.03 to 3.28	1.19	0.16	− 0.48 to 2.85
Indoor as well as outdoor sports	2.17	0.05	− 0.02 to 4.36	2.37	**0.03**	0.19 to 4.55	1.33	0.27	− 1.01 to 3.66
Non-members	− 0.01	0.99	− 1.97 to 1.95	− 0.05	0.96	− 2.00 to 1.91	1.29	0.26	− 0.94 to 3.52
Social support and peers									
Indoor sports	Reference group								
Outdoor sports	1.59	0.06	− 0.07 to 3.26	1.64	0.07	− 0.12 to 3.40	1.04	0.26	− 0.77 to 2.84
Indoor as well as outdoor sports	0.53	0.67	− 1.89 to 2.95	0.70	0.57	− 1.71 to 3.11	− 0.65	0.62	− 3.23 to 1.94
Non-members	− 1.31	0.24	− 3.49 to 0.88	− 1.26	0.26	− 3.44 to 0.91	0.53	0.68	− 1.97 − 3.03
Social acceptance (bullying)									
Indoor sports	Reference group								
Outdoor sports	2.01	**0.03**	0.17 to 3.87	1.80	0.07	− 0.15 to 3.75	1.51	0.14	− 0.50 to 3.52
Indoor as well as outdoor sports	2.62	0.05	− 0.03 to 5.26	2.70	**0.05**	0.05 to 5.35	2.07	0.15	− 0.78 to 4.92
Non-members	0.04	0.97	− 2.32 to 2.40	0.03	0.98	− 2.35 to 2.41	0.83	0.55	− 1.90 to 3.56
School environment									
Indoor sports	Reference group								
Outdoor sports	− 1.14	0.16	− 2.70 to 0.44	− 0.92	0.28	− 2.57 to 0.73	− 1.17	0.18	− 2.87 to 0.53
Indoor as well as outdoor sports	1.40	0.22	− 0.85 to 3.65	1.49	0.20	− 0.77 to 3.75	0.93	0.45	− 1.50 to 3.35
Non-members	− 0.28	0.79	− 2.30 to 1.74	− 0.20	0.84	− 2.24 to 1.83	0.53	0.66	− 1.81 to 2.88
Financial resources									
Indoor sports	Reference group								
Outdoor sports	2.63	**0.001**	1.05 to 4.21	2.56	**0.003**	0.90 to 4.21	2.36	**0.01**	0.65 to 4.07
Indoor as well as outdoor sports	2.26	**0.05**	− 0.01 to 5.53	2.54	**0.03**	0.28 to 4.81	2.11	0.09	− 0.33 to 4.54
Non-members	− 0.73	0.48	− 2.76 to 1.31	− 0.68	0.51	− 2.71 to 1.36	− 0.12	0.92	− 2.46 to 2.22

Significant p’s are in bold

^a^Adjusted for gender, age, BMI, SES and household composition

^b^Adjusted for gender, age, BMI, SES, household composition and frequency of sports participation

^c^Unstandardized regression coefficient

The results presented in Table 5 indicate that frequency of sports participation (added to the models as a continuous variable) was a strong confounder in most of the associations between performing indoor versus outdoor sports and HRQoL.

## Discussion

The present study aimed to explore longitudinal associations between characteristics of sports participation and HRQoL. Of all examined characteristics of sports participation, frequency is by far the most important characteristic that is longitudinally associated with more favourable HRQoL. Membership of a sports club and performing outdoor sports also showed, albeit to a limited extent, longitudinal associations with better HRQoL.

### Membership of a sports club

The observed associations between being a member of a sports club and a more favourable HRQoL for some dimensions are in line with those of previous studies for adults [42, 43], adolescents [11, 44, 45] and children [19–21]. With regard to the physical domain of HRQoL the explanation for the association with the dimension ‘physical well-being’ could be that engaging in sports activities fosters a child’s physical condition [46, 47], which results in higher physical well-being [48, 49]. With respect to the social domain, the longitudinal association of membership of a sports club with better HRQoL for the dimension ‘social support and peers’ does suggest that organized sports activities provide possibilities for children to interact with friends and peers [50]. These interactions facilitate positive social experiences and better social skills leading to a more favourable HRQoL [36, 51].

That only a limited number of associations were found could be explained by the large proportion of children in our sample who were low or moderate sports-active. In this study, it were especially the high sports-active children that showed better HRQoL on almost all dimensions than their peers.

### Frequency of sports participation

Compared to the other examined characteristics of sports participation, higher frequency is by far most longitudinally associated with better HRQoL in this study. With respect to the physical domain, it is likely that sports participation has a ‘feel-good’ effect [52] and affective response [53, 54], which are expressed in, amongst others, a better subjective vitality [55]. In this context, subjective vitality refers to feeling alive and energetic after sports participation and occurs after sports participation during at least 3 days a week [56]. However, an alternative explanation for the longitudinal association between higher frequency and better HRQoL in the physical domain would be that those students who had better HRQoL were more likely to participate in sports activities.

Regarding the psychological domain, based on the Self-determination Theory [57], an explanation for the longitudinal association of frequency of sports participation with better HRQoL for all three dimensions at stake might be that high sports-active children fulfil basic psychological needs for autonomy, competence and relatedness by spending several days at a sports club. Furthermore, high or moderate sports-active children have the possibility to develop better motor skills, which in turn contributes to a positive sports self-perception [58–60]. Finally, the ‘feel-good’ effect of high or moderate active sports participation gives, besides benefits in the physical domain, also a pleasant mood and joy in the psychological domain [56].

With respect to the social domain, the observed longitudinal associations with the dimension ‘home life and parents’ and ‘social support and peers’ suggest that in high sports-active children there is probably a transfer of the various social skills children learned at the sports club to their nearby social environment of parents and friends [61, 62].

### Performing individual versus team sports

The only longitudinal association observed in the present study, performing individual sports being longitudinally associated with better ‘moods and emotion’ compared to performing team sports, is contrary to the conclusion by Vella et al. [21], who found better emotions for children performing team sports. In line with their study, there are many indications that children participating in team sports benefit more from the positive social support, development of social skills and positive peer relationships than children participating in individual sports [36, 51, 63]. However, it may be argued that children performing team sports experience a greater intrinsic pressure [64], a greater pressure from coaches, peers or family [65, 66] and more negative team dynamics [67]. This all can lead to a loss of pleasure due to a high sense of ‘them and us’ [68], and to a loss of autonomy and thus to an increase of boredom or stress [69].

Unlike Vella et al. [21], we did not find a substantial number of longitudinal associations between performing individual versus team sports and better HRQoL. The very small number of significant associations observed in our study may partly be due to the potentially negative aspects of team sports. However, also the fact that children performing individual and children performing team sports experience largely the same group dynamics and social influence in their sport setting at this age could explain the very small difference where the influence on HRQoL is concerned. Although there are indeed different insights in the literature (e.g. Vella et al. [70]), it can be argued that in both types of sports participation, children train together in groups, and when participating in matches also mostly act as a team and experience both the positive and negative aspects of being part of a group event [71, 72].

### Performing indoor versus outdoor sports

In this study, also a limited number of longitudinal associations between performing indoor versus outdoor sports and better HRQoL were observed. This research finding seems, at first sight, to be at odds with studies reporting beneficial effects of being physically active in a natural environment [73]. However, studies in the field of green exercises focus on more forms of physical activity than just sports activities, such as recreational walking, gardening, running and cycling [74], and relate to other environments than sports fields, such as parks, wooded areas and water bodies [75, 76]. The subject of these studies, therefore, differs significantly from the subject of our study.

Our research finding that performing indoor versus outdoor sports was only to a limited extent longitudinally associated with better HRQoL is in line with insights that especially for a child’s HRQoL it is not as relevant whether sports activities are performed in an indoor or an outdoor setting. Hart [77] argues that children consider the environment in which they play sports, primarily as a social meeting place. The location of this social meeting place is of minor importance for them. Reed et al. [61] found that for children green exercises did not create an additional improvement in enjoyment in comparison with outdoor activities in non-green urban areas. Adams et al. [62] described that children’s natural environmental views were not related to their subjective well-being.

However, on the basis of our research, the irrelevance of the distinction between indoor and outdoor sports has to be nuanced for the psychological domain of HRQoL. We observed a weak significant longitudinal association between performing outdoor sports and better ‘self-perception’. This association might be explained by the fact that the sometimes changing weather conditions in the open air (e.g. rain, wind power, and sunshine) give children more opportunities to acquire physical strength and endurance. This might be conducive to their subjective vitality (a positive feeling of aliveness and energy), which in turn may enhance their self-perception [58].

### Strengths and limitation

This is one of the few studies examining longitudinal associations between various characteristics of sports participation and HRQoL in children aged 10–12 years. Until now, only Sánchez-López et al. [19], Tsiros et al. [20] and Vella et al. [21] investigated such associations focusing on children in a slightly younger age group.

Moreover, this is the first study investigating longitudinal associations between characteristics of sports participation and HRQoL in children which used a self-reported measure of HRQoL instead of parent reports. In a healthy population, as is the case in our sample, self-report gives in this age group a better indication of the child’s HRQoL than parent report [78, 79].

While this study presents relevant findings concerning HRQOL in children, there are also some limitations. Although the time spent in sports participation also seems to be relevant for HRQoL [80], the duration of sports participation was not taken into account in our analyses. Based on self-reported information from the children, rather than objectively measured data about physical activity, we were not able to determine the time spent on sports participation in a valid way.

Although the measure instruments comply with the usual requirements of validity and reliability, a well-known limitation of a longitudinal observational design as applied in our study concerns the impossibility to establish possible causal relationships, which is a limitation that in general applies to all non-experimental studies. Only statistical associations could be ascertained.

Furthermore, the risk of possible bias due to the applied sampling strategy did not materialize. The group of children included in the study was comparable with the general Dutch population of primary school children aged 10–12 important characteristics such as BMI, SES and membership of a sports club for children.

The group of children in the follow-up differed to a small extent from the dropouts with respect to frequency of sports participation and the HRQoL dimension ‘physical well-being’. The frequency of sports participation of the dropouts was significant higher and their HRQoL on the dimension ‘physical well-being’ was significantly lower. These differences may have led to a slight underestimation of some longitudinal associations for frequency of sports participation and a slight overestimation for the HRQoL dimension physical well-being.

Finally, a multiple testing problem could arise because many statistical analyses have been performed. In order to cope with this, we have not focused so much on separate, significant associations in our conclusions, but mainly focused our attention to the overall picture per HRQoL domain.

### Future research

In future research, other characteristics of sports participation like duration and intensity could also be taken into account. Furthermore, in addition to self-report data, information from accelerometers could be used to increase validity. Finally, future research preferably takes place in the form of an intervention in order to find out whether the statistical associations observed in our study are of a causal nature.

## Conclusion

Better HRQoL is to a considerable extent longitudinally associated with higher frequency of sports participation and only to a limited extent longitudinally associated with performing individual sports and performing outdoor sports. Frequency of sports participation seems to be much more relevant for a child’s HRQoL than the kind of sport(s) in which the child participates. Therefore, the choice for a sport that the child can and likes to do with a relatively high frequency is presumably more important than the choice for a certain type of sport.

## Electronic supplementary material

Below is the link to the electronic supplementary material.

Supplementary material 1 (DOCX 53 kb)
