# Supplementary material for: Sports participation and health-related quality of life: a longitudinal observational study in children
Source: Qual Life Res. 2019 Jun 3;28(9):2453–69. doi: 10.1007/s11136-019-02219-4 (PMC6698265; doi:10.1007/s11136-019-02219-4)
Supplement: Supplementary file 1 — Supplementary material 1 (DOCX 53 kb) [file 11136_2019_2219_MOESM1_ESM.docx]

**Journal:** Quality of Life Research

**Sports participation and health-related quality of life: A longitudinal observational study in children**

Janet Moeijes, Jooske T. van Busschbach, Ruud J. Bosscher, and Jos. W. R. Twisk

Janet Moeijes (corresponding author)
Department of Human Movement and Education, Windesheim University of Applied Sciences, Campus 2-6, Zwolle, 8017CA, The Netherlands
[j.moeijes@windesheim.nl](mailto:j.moeijes@windesheim.nl)

Amsterdam UMC, Vrije Universiteit Amsterdam, Department of Epidemiology and Biostatistics, Amsterdam Public Health research institute, Van der Boechorststraat 7, 1081BT Amsterdam, The Netherlands

**Additional file 1: Flowchart gathering schools**

Wave 1

Course 2011-2012

72 schools approached

49 involved

Excluded 3 schools because no fourth grade children included

Wave 2

Course 2012-2013

23 new schools approached

Course 2012-2013

29 schools approached for the 2nd time

13 new schools involved

24 schools involved for second measurement

Excluded 6 schools because only grade 5 children

Course 2013-2014

21 new schools approached

Course 2013-2014

7 schools approached for 2nd measurement

Course 2013-2014

8 schools approached again

Wave 3

11 new schools involved

5 schools involved

8 schools involved

Longitudinal sample of 29 schools

*Figure S2.* Flowchart regarding the gathering of schools

**Additional file 2: Validation sports participation questions**

The questions from the Movement and Sports Monitor Questionnaire Youth aged 8-12 year (MSMQ) with which children's sport participation was determined were as follows.

1. Are you a member of a sports club?

□ Yes

□ No [proceed to question 28]

2. What kind of sports do you play at a sports club?

You may name several sports.

□ 1…………………………………….

□ 2…………………………………….

□ 3…………………………………….

□ 4…………………………………….

3a. How many times a week do you have a training?

Enter the sport on the line and answer the question after this.

……….. SPORT 1 ………. time(s) a week

………. SPORT 2 ………. time(s) a week

………. SPORT 3 ………. time(s) a week

………. SPORT 4 ………. time(s) a week

3b. How many times a week do you have a match?

[If you do not have matches, proceed to question 25].

Enter the sport on the line and answer the question after this.

………. SPORT 1 ………. match(es) a week

………. SPORT 2 ………. match(es) a week

………. SPORT 3 ………. match(es) a week

………. SPORT 4 ………. match(es) a week

3c. How many times each month do you have a tournament?

[If you do not have any tournament, proceed to question 28].

Enter the sport on the line and answer the question after this.

………. SPORT 1 ………. tournaments per month

………. SPORT 2 ………. tournaments per month

………. SPORT 3 ………. tournaments per month

………. SPORT 4 ………. tournaments per month

We ensured the validity of the abovementioned sports participation questions in three ways: (i) a pilot study among primary school children, (ii) an expert meeting and individual experts consultations (with respect to face validity) and (iii) a comparison of some research findings about children’s sports participation with national data.

Pilot study among primary school children

We did a pilot study at three elementary schools with in total 43 children involved. On each school we formed a focus group of children to check whether the questions about sports participation were understood by the children. The focus group consisted of five to six children stratified by gender and school grade. In the focus group, children discussed different aspects of their sports participation. The principal investigator, the first author of the article, asked the children for their experiences with the three sports participation questions of the MSMQ.

The questions concerned membership of a sports club, frequency of sports participation, and duration of sports participation. Children positively evaluated the question about membership of a sports club. They missed, however, a question about which sports they were practicing. Therefore, we added a question about this issue. Furthermore, children found it too difficult to immediately estimate the total frequency of training activities and matches. In response to their feedback, we split the frequency question into a question about the frequency of training activities, a question about the frequency of matches, and a question about tournaments. Finally, the question about the total duration of their sports activities appeared to be unclear for children, especially for children performing individual sports. Therefore, we deleted this question.

In the last step of the pilot study, the modified sports questions of the MSMQ were offered to ten fourth and fifth-grade elementary school children. The children were from another primary school than the children who participated in the focus group study. They were well able to answer the questions.

Expert meeting and individual expert consultations (with respect to face validity)

After the pilot study we conducted a meeting with five experts in the field of sports participation, and four individual expert consultations. The experts were asked to evaluate the clearness of the modified questions for children aged 10-12 years and the suitability of the questions to measure certain aspects of sports participation in children.

The experts had to assess the questions on the basis of the following two criteria: 1. Are the questions sufficiently clear for children aged 10 to 12 (group 7 and 8 of primary school)? In other words: do children understand the contents of the questions?; and 2. Do the questions measure what they intend to measure? In other words: Do the questions really qualify as a member of a sports club, on which sport or sports a child is and the frequency of the training sessions, competitions and tournaments per sport?

Based on the feedback of the experts, it was concluded that the validity of the sports questions was sufficient.

Comparison of research findings about sports participation with national data

Construct validity has not yet been established. Our argument for this is that we did not intend to measure a construct. Our intention was to measure several separate aspects of sports participation, including membership sports club and frequency of sports participation. Our findings about membership and frequency appeared to be consistent with the results based on a large representative sample of children of the same age in the Netherlands (Kenniscentrum Sport, 2017; Tiessen-Raaphorst & Van den Dool, 2015)
